# *Kras* mutation rate precisely orchestrates ductal derived pancreatic intraepithelial neoplasia and pancreatic cancer

**DOI:** 10.1038/s41374-020-00490-5

**Published:** 2020-10-02

**Authors:** Kanchan Singh, Melissa Pruski, Rachael Bland, Mamoun Younes, Sushovan Guha, Nirav Thosani, Anirban Maitra, Brooks D. Cash, Florencia McAllister, Craig D. Logsdon, Jeffrey T. Chang, Jennifer M. Bailey-Lundberg

**Affiliations:** 1 Division of Gastroenterology, Hepatology and Nutrition, Department of Internal Medicine, McGovern Medical School, The University of Texas Health Science Center, Houston, TX 77030 USA; 2 Kings College London, Department of Pharmacology, London, UK; 3 Department of Pathology and Laboratory Medicine, McGovern Medical School, The University of Texas Health Science Center, Houston, TX 77030 USA; 4 Department of Translational Molecular Pathology, Division of Pathology and Laboratory Medicine, The University of Texas MD Anderson Cancer Center, Houston, TX USA; 5 Department of Gastrointestinal Medical Oncology, The University of Texas MD Anderson Cancer Center, Houston, TX USA; 6 Department of Cancer Biology, The University of Texas MD Anderson Cancer Center, Houston, TX USA; 7 Department of Integrative Biology and Pharmacology, McGovern Medical School, The University of Texas Health Science Center, Houston, TX 77030 USA; 8 Department of Anesthesiology, McGovern Medical School, The University of Texas Health Science Center, Houston, TX 77030 USA

**Keywords:** Cancer models, Mechanisms of disease

## Abstract

Pancreatic ductal adenocarcinoma (PDAC) is the third leading cause of cancer-related death in the United States. Despite the high prevalence of *Kras* mutations in pancreatic cancer patients, murine models expressing the oncogenic mutant *Kras* (*Kras*^*mut*^) in mature pancreatic cells develop PDAC at a low frequency. Independent of cell of origin, a second genetic hit (loss of tumor suppressor *TP53* or *PTEN*) is important for development of PDAC in mice. We hypothesized ectopic expression and elevated levels of oncogenic mutant *Kras* would promote PanIN arising in pancreatic ducts. To test our hypothesis, the significance of elevating levels of K-Ras and Ras activity has been explored by expression of a CAG driven *LGSL-Kras*^*G12V*^ allele (*cKras*) in pancreatic ducts, which promotes ectopic *Kras* expression. We predicted expression of *cKras* in pancreatic ducts would generate neoplasia and PDAC. To test our hypothesis, we employed tamoxifen dependent CreER^T2^ mediated recombination. *Hnf1b:CreER*^*T2*^*;Kras*^*G12V*^ (*cKras*^*Hnf1b/+*^) mice received 1 (Low), 5 (Mod) or 10 (High) mg per 20 g body weight to recombine *cKras* in low (*cKras*^*Low*^), moderate (*cKras*^*Mod*^), and high (c*Kras*^*High*^) percentages of pancreatic ducts. Our histologic analysis revealed poorly differentiated aggressive tumors in *cKras*^*High*^ mice. *cKras*^*Mod*^ mice had grades of Pancreatic Intraepithelial Neoplasia (PanIN), recapitulating early and advanced PanIN observed in human PDAC. Proteomics analysis revealed significant differences in PTEN/AKT and MAPK pathways between wild type, *cKras*^*Low*^, *cKras*^*Mod*^, and *cKras*^*High*^ mice. In conclusion, in this study, we provide evidence that ectopic expression of oncogenic mutant K-Ras in pancreatic ducts generates early and late PanIN as well as PDAC. This Ras rheostat model provides evidence that AKT signaling is an important early driver of invasive ductal derived PDAC.

## Summary

In this study, the authors provide evidence that ectopic expression of oncogenic mutant *Kras* in pancreatic ducts generates early and late (PanIN) and pancreatic ductal adenocarcinoma (PDAC) . They characterized this Ras rheostat model which reveals elevated *Kras* mutation frequency and loss of PTEN are important drivers of PanIN and invasive ductal derived PDAC.

## Introduction

Cell of origin differences  have  been shown to promote molecular heterogeneity in a number of malignancies [1, 2]. Pancreatic ductal adenocarcinoma (PDAC) displays remarkable epithelial and stromal heterogeneity and both genetic and pathologic heterogeneity have been described indicating multiple cells of origin may contribute to this aggressive malignancy [3–13]. PDAC is aggressive and diagnosis before cancer cells have locally invaded or metastasized is uncommon. Understanding the biology of how exocrine pancreatic cells transform to cancer is important for development of targeted therapeutic approaches for prevention of aggressive disease. PDAC is preceded by precursor lesions postulated to arise from pancreatic exocrine epithelium. Two major precursor lesions include Pancreatic Intraepithelial Neoplasia (PanIN) and Intraductal Papillary Mucinous Neoplasia (IPMN). PanIN are most frequently observed in resected PDAC and are thought to precede development of invasive adenocarcinoma [14, 15]. A well-established autochthonous mouse model has shown PanIN arise in the setting of physiologic expression of *Kras*^*G12D*^ in Pdx1 (or Ptf1a/p48) expressing multipotent pancreatic progenitors. In this model, at 9 months of age, mice display the full histological spectrum of early and advanced PanIN lesions [16]. In contrast, adult pancreatic exocrine cells are refractory to oncogenic *Kras*^*mut*^ induced invasive carcinoma when *Kras*^*G12D*^ is expressed under endogenous promoter elements unless there is induced inflammation or concomitant loss of a tumor suppressor gene [17–22].

Somatic mutation in codon 12 of *KRAS* is the most common point mutation in PDAC [23]. Although *KRAS* is mutated in more than 93% of sequenced human PDAC and is described as the initiating genetic event for cancer formation, studies using genetically engineered mouse models reveal that physiologic expression of oncogenic *Kras*^*mut*^ alone can promote PanIN, but it is rarely sufficient for tumor development in pancreatic exocrine cells [4, 17, 18, 24–27]. Mouse models have shown oncogenic *Kras*^*mut*^ can promote senescence in healthy cells, which is overcome by mutations in or genetic deletion of tumor suppressor genes [28–32]. These models are informative regarding development of oncogenic *Kras*^*mut*^ driven malignancies as *KRAS*`mutations occur, even in the absence of neoplastic pancreata [33].

In this study, our goal was to determine if expression of *cKras* would promote IPMN, PanIN, or PDAC in ductal pancreatic epithelium. We wanted to test the distinct pathologies this allele could promote, as pancreatic ducts have been thought to be the cellular origin of PDAC due to the predominant ductal and glandular histology of PDAC[14, 34]. Furthermore, recent analysis of somatic variants in human PDAC and precursor lesions has shown a multi-step progression in which advanced PanIN are a single neoplasm that colonize the ductal system [35]. Despite this presumption, early studies using genetic mouse models indicated acinar cells are a possible cellular origin [36–38]. CreER mediated expression of oncogenic *Kras*^*mut*^ in mature, adult acinar cells led to PanIN lesions that transformed to PDAC when oncogenic *Kras*^*mut*^ was expressed in combination with chronic inflammation, high fat diet, or mutant tumor suppressor genes [17, 21, 39, 40]. Recently, pancreatic duct ligation was used to model chronic obstructive pancreatitis in the context of cell of origin. In this model, expression of oncogenic *Kras*^*mut*^ and pancreatic duct ligation resulted in PDAC in ductal, but not acinar cells [41]. In addition, recent studies have shown that mutations in or genetic loss of *Trp53* or *PTEN* results in ductal or acinar derived PDAC [4, 17, 22, 41, 42]. These models are important as human PDAC subtypes, which may arise from divergent cells of origin, have been characterized and indicate differences in prognosis based on histologic, genetic, and RNA sequencing data [6, 8, 23, 43, 44].

The overall hypothesis guiding this work is mouse pancreatic ductal cells, which are otherwise refractory to transformation under conditions of physiologic oncogenic *Kras*^*mut*^ expression, will become morphologically distinct PanIN or IPMN when expressing *cKras*. We predict if *cKras* is able to generate neoplastic and invasive PDAC that this model will provide important context and a new model to study molecular requirements for ductal derived PDAC. As previously reported [19]*, cKras* is engineered following a ubiquitously expressed CAG promoter blocked by proximal insertion of a loxP-green fluorescent protein (GFP)-STOP-loxP cassette, which allows for lineage tracing. In this model, intraperitoneal injection of tamoxifen promotes CreER dependent recombination of loxP sites flanking a STOP cassette and GFP allele; which results in expression of *cKras* allele and loss of GFP [19, 45]. As we have previously worked with the *Hnf1b:CreER* allele, in initial experiments, 6–8 week old mice were given 10 mg of tamoxifen. We observed very aggressive lethality in these mice and thus titrated our tamoxifen dose, which led to generation of this Ras rheostat model of ductal derived PanIN and PDAC. We predict this model is relevant to what has been shown in sequencing of human PanIN, wherein while >90% of sequenced PanIN have mutations in *KRAS*, quantification of mean *KRAS* mutations per PanIN significantly increases in progression from early to advanced PanIN, indicating increasing numbers of pancreatic ductal cells with oncogenic *KRAS* is a method by which cells elevate K-Ras oncogenic signaling pathways important for transformation [33].

## Results

### Tamoxifen dosage directly correlates with percentage of recombined pancreatic ducts

**Fig. 1:**
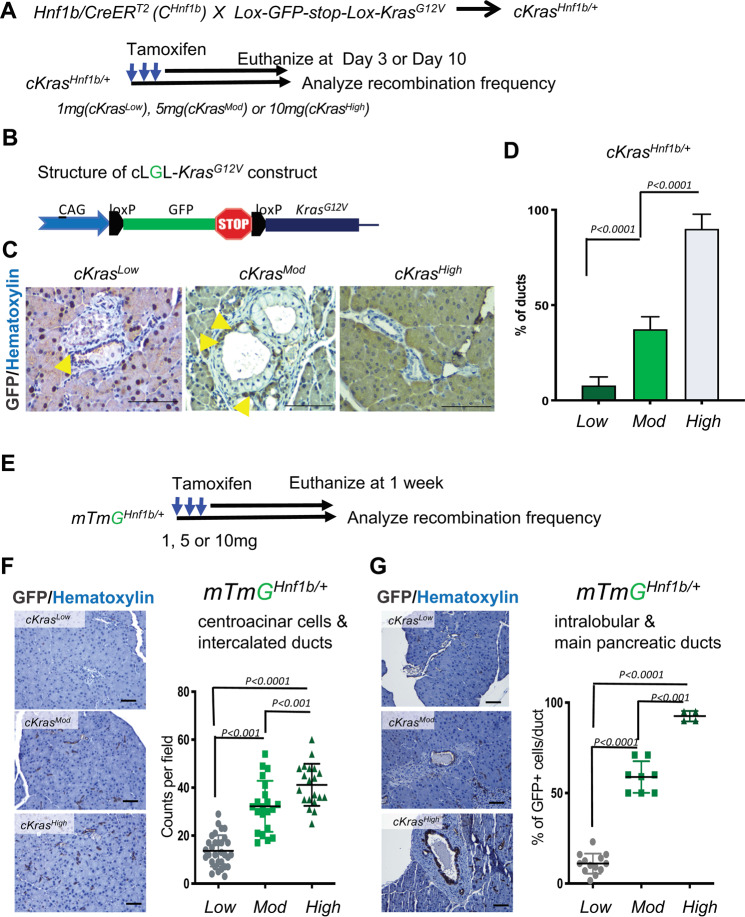
Tamoxifen dosage directly correlates with recombination frequency in pancreatic ducts. **a** Schematic showing the experimental strategy to lineage trace Hnf1b+ duct cells in the pancreas of *cKras*^*Hnf1b/+*^ mice for analysis of tamoxifen mediated CreER^T2^ recombination. At an age of 6–8 weeks, mice received 1 mg (*cKras*^*Low*^), 5 mg (*cKras*^*Mod*^), or 10 mg (*cKras*^*High*^) of tamoxifen and were analyzed at both early and late timepoints. **b** Structure of the *cLGL-Kras*^*G12V*^ ectopic expression transgene. *cKras* was engineered following a human CMV and chicken-β-actin chimeric promoter (CAG) and blocked by the proximal insertion of a loxP-green fluorescent protein (GFP)-Stop-loxP cassette (**c**) Lineage tracing shows that PDAC precursors (PanIN and micropapillary lesions) and associated PDAC arise in pancreatic ducts of mice. Recombination in ducts depends on tamoxifen dosage (yellow arrows show nonrecombined ducts in *cKras*^*Low*^ and *cKras*^*Mod*^ at end point and *cKras*^*High*^ mice at day3 post tamoxifen). **d** Quantification of percentage of ductal cells recombined in *cKras*^*Low*^, *cKras*^*Mod*^, and *cKras*^*High*^ mice. We observed a significant increase in recombination comparing *cKras*^*Low*^ (7.8 ± 4.5%) to *cKras*^*Mod*^ (37.3 ± 6.5%) and *cKras*^*Mod*^ to *cKras*^*High*^ (90 ± 7.62%). **e** Schematic showing the experimental strategy to lineage trace Hnf1b+ duct cells in the pancreas of *mTmG*^*Hnf1b/+*^ for analyzing tamoxifen mediated Cre recombination. Mice received 1, 5, and 10 mg of tamoxifen and were analyzed at 1 week. **f** and **g** IHC staining for GFP and quantification of recombined centroacinar and intercalated ducts. GFP immunolabeling significantly increased in centroacinar and intercalated ducts in *mTmG*^*Hnf1b/+ High*^ mice with an average count of 41/field compared to *mTmG*^*Hnf1b/+ Low*^ (13/field) or *mTmG*^*Hnf1b/+ Mod*^ (32/field) mice. *mTmG*^*Hnf1b/+ Mod*^ mice had significantly higher numbers of recombined centroacinar cells and intercalated ducts than *mTmG*^*Hnf1b/+ Low*^ mice (*P* < 0.001). **g** When analyzing recombination in main pancreatic and intralobular ducts, we observed a significant increase in *mTmG*^*Hnf1b/+ Mod*^ (58% ducts/field) and *mTmG*^*Hnf1b/+ High*^ (92% of ducts/field) mice compared to *mTmG*^*Hnf1b/+ Low*^ mice (11% ducts/field). Statistical analysis was performed using a Two-way Anova in Prism GraphPad software.

We examined tamoxifen dependent recombination of *cKras* allele. Figure 1a shows a schematic for generation of c*Kras*^*Hnf1b/+*^ mice and Fig. 1b is a graphic of the inducible *cKras* allele. When allelic recombination of loxP sites occurs, recombined cells lose GFP. Thus, initial methodology used to study occurrence of recombination in pancreatic ducts was loss of GFP. Paraffin embedded sections from mice euthanized at both early and late time points were used for analysis. Immunohistochemistry (IHC) staining for GFP was used to detect the presence or absence of recombination. For all experiments, mice were administered tamoxifen at an age of 6–8 weeks. We observed a significant increase in recombination (loss of GFP) in ductal cells as a function of tamoxifen dose. Non-recombined GFP+ cells (Fig. 1c, yellow arrows) are present in *cKras*^*Low*^ and *cKras*^*Mod*^ pancreatic sections. In *cKras*^*High*^ mice, tissue sections from early time points were evaluated as these mice rapidly developed invasive PDAC, which compromised our lineage tracing methods (Supplementary Fig. 1). We quantified recombined epithelium (number of GFP- ducts/total number of ducts) for all models, which revealed a significant increase in recombination comparing *cKras*^*Low*^ (7.8 ± 4.5%) to *cKras*^*Mod*^ (37.3 ± 6.5%) and *cKras*^*Mod*^ to *cKras*^*High*^ (90 ± 7.62%) (Fig. 1d). As a second method, we used the *loxP-mTdTomato-loxP-mGFP (Rosa*^*mTmG*^) [46] reporter allele crossed to *Hnf1b:CreERT2* animals to study recombination (gain of GFP expression) in pancreatic ducts as a function of tamoxifen dose (*mTmG*^*Hnf1b/+*^) (Fig. 1e). These mice were euthanized 1 week after they were given tamoxifen. We used immunohistochemistry and stained *mTmG*^*Hnf1b/+Low*^*, mTmG*^*Hnf1b/+Mod*^, and *mTmG*^*Hnf1b/+High*^ murine pancreatic sections with GFP and quantified recombined ducts (Fig. 1f, g). We observed higher numbers of GFP + ducts which indicated increased recombination in centroacinar and intercalated ducts in *mTmG*^*Hnf1b/+Hig*h^ mice with an average count of 41/field compared to *mTmG*^*Hnf1b/+Low*^ (average count of 13/field) or *mTmG*^*Hnf1b/+Mod*^ (average count of 32/field) (Fig. 1f). *mTmG*^*Hnf1b/+Mod*^ mice also had significantly higher numbers of recombined centroacinar cells and intercalated ducts than *mTmG*^*Hnf1b/+Low*^ mice (Fig. 1f). When analyzing recombination in main pancreatic and intralobular ducts, we observed a significant increase in *mTmG*^*Hnf1b/+Mod*^ (58% ducts/field) and *mTmG*^*Hnf1b/+High*^ (92% of ducts/field) mice compared to *mTmG*^*Hnf1b/+Low*^ mice (11% ducts/field) (Fig. 1g).

### *cKras* recombination in higher percentages of ductal cells directly affects survival

**Fig. 2:**
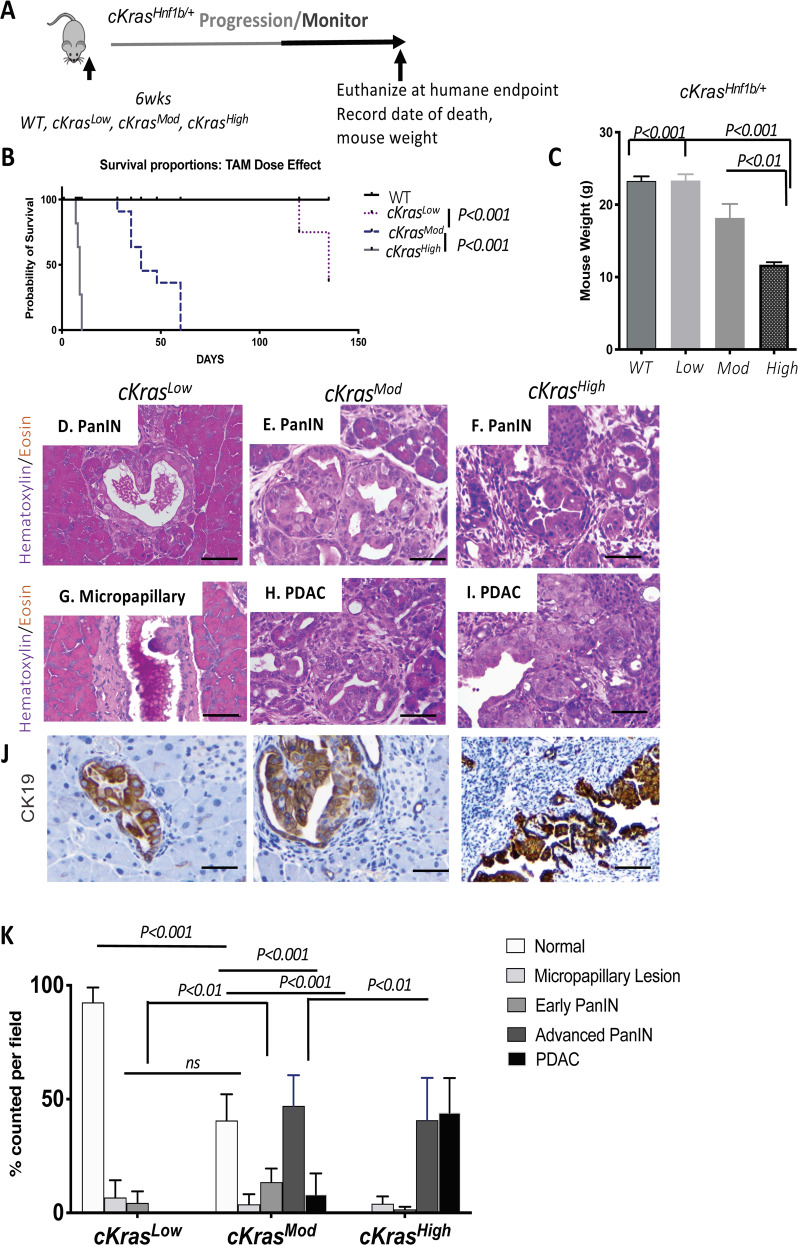
Pathologic outcomes of increasing the percentage of pancreatic ductal cells expressing *cKras*. **a** Schematic of experimental plan to study the effect of titrating tamoxifen given to mice to study recombination efficiency and effects on pancreatic histology. **b** Increased dosage of tamoxifen significantly decreases survival of *cKras*^*Hnf1b/+*^ mice. *cKras*^*High*^ mice survive an average of 10 days, *cKras*^*Mod*^ mice survive an average of 42 days and 50% of *cKras*^*Low*^ mice are still alive 138 days after administered tamoxifen. The other 50% had to be euthanized. **c** *cKras*^*High*^ and *cKras*^*Mod*^ have a significant reduction in body weight at time of death (*P* < 0.001) compared to littermate control weight and *cKras*^*Low*^ mice at time of death. Statistical analysis was evaluated using a student’s *t* test. **d**–**i** Representative H&E images and CK19 IHC of pancreas from *cKras*^*Low*^, *cKras*^*Mod*^ and *cKras*^*High*^ mice. **d** *cKras*^*Low*^ mice show limited early PanIN and (**g**) Micropapillary lesions. (**e**) *cKras*^*Mod*^ mice have early and advanced PanIN and (**h**) PDAC whereas *cKras*^*High*^ mice have predominantly advanced PanIN (**f**) and PDAC(I). (**j**) CK19 labeling of ductal lesions in *cKras*^*Low*^, *cKras*^*Mod*^ and *cKras*^*High*^ mice. **k** Quantification of normal ducts, micropapillary lesions, early PanIN, advanced PanIN, and PDAC as a consequence of tamoxifen dosage. Lesions were counted and percentages were calculated using total ducts as the denominator. A two-way Anova using Prism Graphpad software evaluated statistical significance. An *n* = 8 mice per tamoxifen dosage was used for pathologic analysis. Scale bars are 50 µM.

Endogenous expression of oncogenic *Kras* alone has a low penetrance of generating PanIN and PDAC in mature adult pancreatic ducts [17, 24]; thus, we examined if ectopic expression *cKras* would transform ductal cells*. cKras*^*High*^ mice aged 6–8 weeks were administered 10 mg of tamoxifen. (Fig. 2a) and within 10–14 days, we noted severe weight loss and lethality in 100% of injected animals (Fig. 2b, c). As such a drastic effect on survival was observed, we analyzed pancreatic head, body, and tail histology on paraffin embedded sections which revealed invasive PDAC throughout the entire pancreatic parenchyma (Supplementary Fig. 1 & 2).

We hypothesized varying levels of tamoxifen would be proportional to changes in histologic outcomes and survival. To test our hypothesis, *cKras*^*Low*^ and *cKras*^*Mod*^ mice were injected at the age of 6–8 weeks (Fig. 2a). Mice were monitored for evidence of disease progression. *cKras*^*Mod*^ mice survived on average 6 weeks and 50% of *cKras*^*Low*^ mice remained healthy and were euthanized at 16 weeks for histologic analysis (Fig. 2b). Upon necropsy and histologic examination of euthanized *cKras*^*Low*^ mice, we observed adenoma formation in the lungs (Supplementary Fig. 2). In the lungs of *cKras*^*Mod*^ mice, we observed adenoma and adenocarcinoma and in *cKras*^*High*^ mice, we observed severe inflammation in interstitial spaces and alveolar lining but no adenoma or adenocarcinoma (Supplementary Fig. 2B). These results are similar to pathologies we recently published [47]. We measured mouse body weight at time of necropsy and noted significant weight loss at time of death in *cKras*^*Mod*^ and *cKras*^*High*^ mice compared to *cKras*^*Low*^ mice and littermate controls that received no tamoxifen (Fig. 2c).

### Levels of *cKras* directly modulate early and advanced PanIN and PDAC

**Fig. 3:**
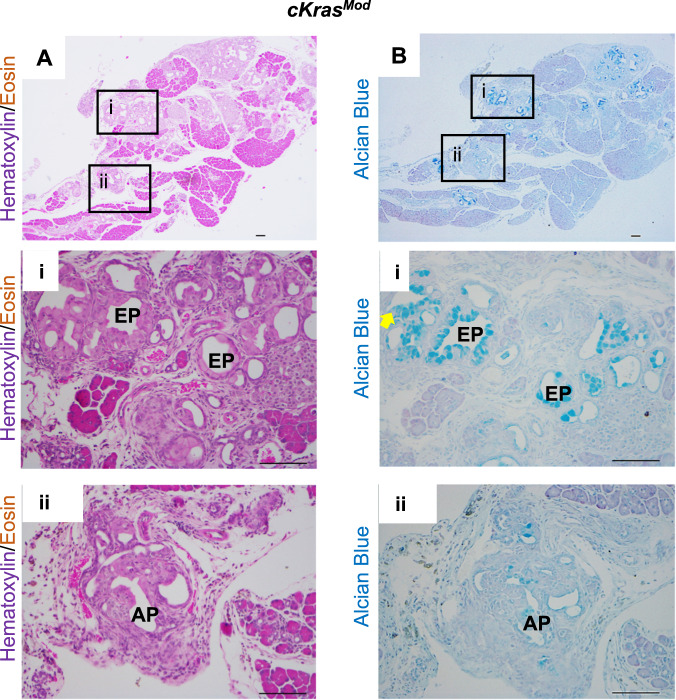
*cKras*^*Mod*^ expression in murine pancreata results in full spectrum of PanIN and PDAC. **a** H&E images of early (i) and advanced (ii) PanIN lesions arising in pancreatic ducts of *cKras*^*Mod*^ mice. Early PanIN (EP) have columnar epithelium and mucin and papillary outgrowths (ii) advanced PanIN (AP) occasionally have loss of basement membrane. **b** Serial section panel is Alcian blue staining of tissue shown in (**a**). Scale bars are 50 µM.

**Fig. 4:**
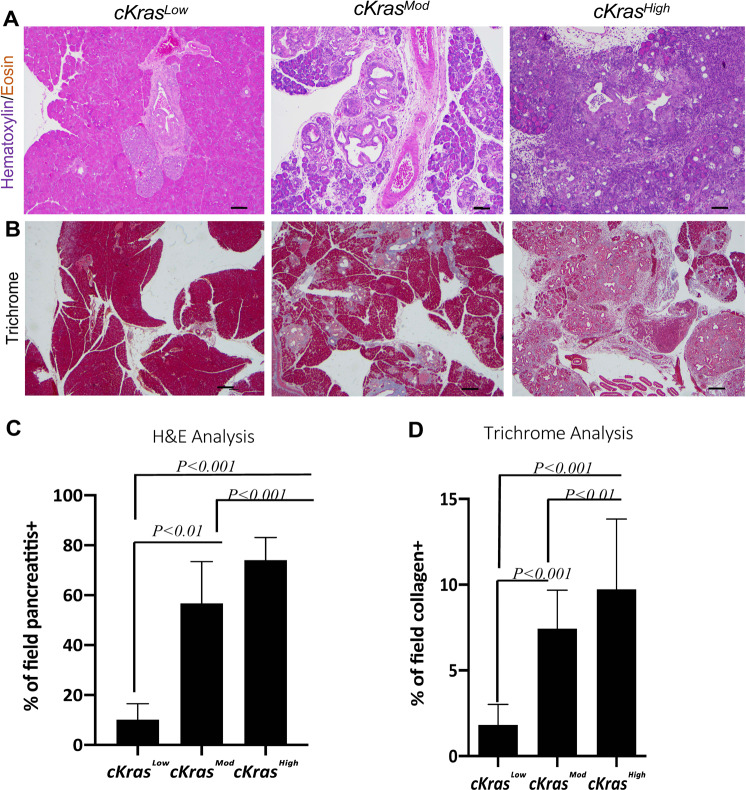
Pancreatitis and fibrostroma are elevated in *cKras*^*Mod*^ and *cKras*^*High*^ pancreata. **a** Representative H&E images of *cKras*^*Low*^, *cKras*^*Mod*^, and *cKras*^*High*^ mice showing increasing pancreatitis surrounding the ductal lesions with increased *Kras* expression. Increased lymphocyte infiltration can be noted. **b** Trichrome staining of pancreas from *cKras*^*Low*^, *cKras*^*Mod*^, and *cKras*^*High*^ showing increased fibrillar collagen. **c** and **d** quantification of pancreatitis and fibrillar collagen per field in *cKras*^*Low*^, *cKras*^*Mod*^, and *cKras*^*High*^ mice. 5 fields per slide were selected for quantification and 5 mice were analyzed per tamoxifen dose. Analysis of pancreatitis area and collagen+ stroma were completed using imageJ software with color deconvolution plugin (Supplementary Fig. 5). Statistical analysis was done using a one-way Anova and Prism Graphpad software.

To characterize the histology of *cKras* mice, we used H&E staining, cytokeratin 19 (CK19) immunolabeling, and alcian blue to quantify the occurrence and abundance of early and advanced PanIN lesions (Figs. 2d and 3). For quantification, we counted the number of each type of ductal lesion and divided this number by the total number of ducts per field. For *cKras*^*Low*^ mice, 92.5 ± 6.5% of pancreas had normal ductal histology with evidence of proliferative ductal epithelium (Supplementary Fig. 3A and Fig. 4B), low abundance intra ductal micropapillary lesions (Fig. 2g, 5.6 ± 9.1%) and early PanIN (4.37 ± 0.5, Fig. 2d). In *cKras*^*Mod*^ mice, we observed early and advanced PanIN and PDAC (Fig. 2e, h). We observed 38.6 ± 11.4% normal ducts and early PanIN occupied 13.5 ± 6% of pancreatic ductal tissue. Advanced PanIN occupied on average 41 ± 13.5% of ductal epithelium and PDAC was observed in 8.1 ± 9.23% of ducts. All *cKras*^*High*^ pancreata (15/15) had no remaining normal ducts and early PanIN were infrequently observed (3.25 ± 2.4%) as these mice had predominantly advanced PanIN (Figs 2f, 40.75 ± 18.59%) and PDAC (Fig. 2i, 44.1 ± 15.1%). To determine if *cKras*^*Mod*^ mice had early or advanced PanIN, we stained with H&E and alcian blue, which confirmed mucinous PanIN (Fig. 3a, b). To confirm the absence of early PanIN in *cKras*^*High*^ mice, we analyzed alcian blue staining in pancreata of mice at earlier time points (days 5 and day 7 post tamoxifen injection), which revealed only advanced PanIN lesions (Supplementary Fig. 3A, B). As elevated Ras signaling has been shown to regulate senescence in ductal epithelium, we stained our sections for p21 and PCNA, which revealed no expression of p21 in ducts and abundant staining for PCNA (Supplementary Fig. 4). These data are interesting as we previously observed expression of p21 in pancreatic ducts expressing *Kras*^*G12D*^ under regulation of endogenous promoter elements [17].

Histologic evaluation of *cKras*^*Mod*^ and *cKras*^*High*^ pancreata revealed pancreatitis and stromal composition near areas of PanIN and PDAC arising in ductal epithelium (Fig. 4). To determine if significant differences in pancreatitis and stroma were evident, we calculated percentage of pancreatitis or collagen + fibrostroma in five mice per dosage in *cKras*^*Mod*^ and *cKras*^*High*^ sections compared to *cKras*^*Low*^ sections (Fig. 4a, b). To accurately determine pancreatitis and stromal composition, we used ImageJ software and color deconvolution plugin to analyze percentage of pancreatitis (or collagen+) area divided by total area of each section (Supplementary Fig. 5 and Fig. 4C, D). Ductal neoplasia in *cKras*^*Mod*^ and *cKras*^*High*^ mice was associated with significantly increased pancreatitis and collagen positive fibrostroma (Fig. 4c, d). In these sections, we also observed infiltration of CD68 + macrophage and CD45 + lymphocytes, indicating in this model, infiltrating immune cells are also a component of stroma (Supplementary Fig. 4).

### Increased recombination frequency correlates with total Ras in pancreatic tissue

**Fig. 5:**
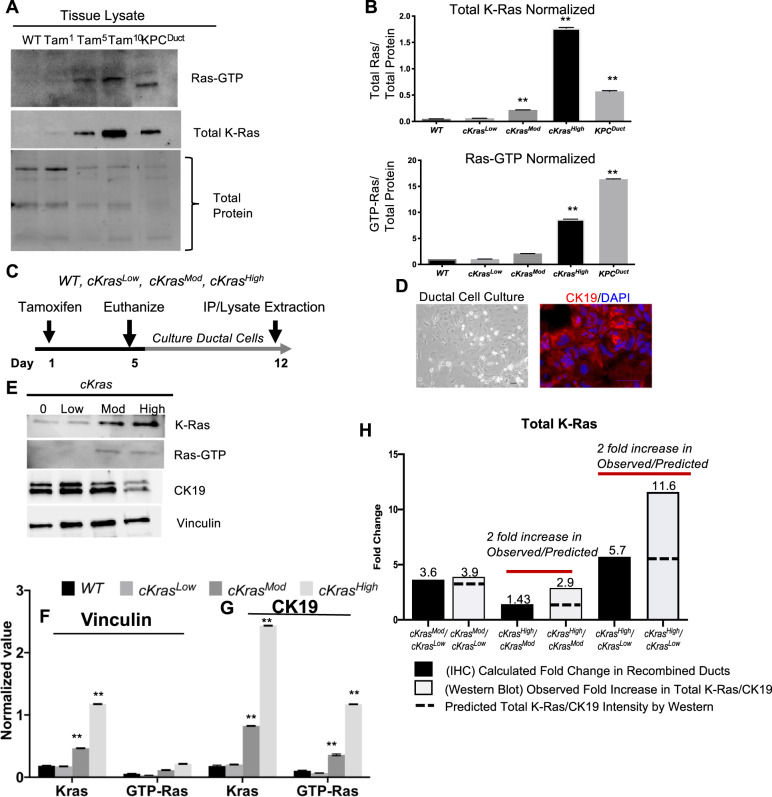
Tamoxifen dose is directly related to total Ras levels and signaling. **a** Lysates from wild-type pancreas (Lane1), *cKras*^*Low*^*, cKras*^*Mod*^, and *cKras*^*High*^ murine pancreas (lanes 2,3,4) were affinity precipitated with Raf RBD agarose and subjected to immunoblot analysis with anti-Ras antibody. **b** Quantification of Total Ras protein and RAS-GTP to total protein confirmed a significant increase in Ras expression and GTP bound Ras with increased tamoxifen dosage. **c** Experimental plan to establish ductal cell culture from *cKras*^*Hnf1b/+*^ mice. **d** Brightfield image of primary pancreatic duct culture and adjacent panel showing CK19 IF labeling confirming ducts. **e **Western blot analysis of lysates from primary ducts cultured from WT, *cKras*^*Low*^*, cKras*^*Mod*^, and *cKras*^*High*^ murine pancreas four days after tamoxifen administration. **f** Normalization of western blot image. Normalization of protein expression was done in triplicate using vinculin as the control for ductal cell culture expression analysis. **g** Normalization of total K-Ras and GTP bound Ras using CK19 as epithelial marker control for duct cells indicates increased K-Ras expression per cells with increased tamoxifen dose. **h** Comparison of K-Ras expression predicted vs calculated based on IHC and western analysis indicating increased K-Ras expression per cell with increased tamoxifen dosage. Statistical analysis was done using a Two-way Anova in Prism GraphPad.

As we observed such drastic differences in histology when comparing *cKras*^*Low*^*, cKras*^*Mod*^, and *cKras*^*High*^ mice, we wanted to examine levels of total K-Ras and Ras-GTP in this ductal cell of origin PanIN and PDAC system. We performed a Ras pull down assay to measure levels of Ras activity in pancreata from *cKras*^Low^, *cKras*^Mod^, and *cKras*^High^ mice and directly compared the levels of Ras-GTP to a previous ductal cell of origin model we published by expressing Kras^G12D^ and Trp53^R175H^ (*KPC*^*Duct*^) [17]. Western blot analysis and normalization to total protein in these pancreata indicated significantly higher levels of Total K-Ras and Ras-GTP in *cKras*^*Mod*^*, cKras*^*High*^, and *KPC*^*Duct*^ tissue compared to *cKras*^*Low*^ (Fig. 5a, b). These data are similar to previous publications showing loss of Trp53 or gain of function mutations in Trp53 can elevate Ras-GTP and promote aggressive PDAC in mice [19, 48].

### In vitro duct cultures reveal *cKras*^*Mod*^ and *cKras*^*High*^ ducts have significantly higher GTP bound Ras

To further study effects of increased *cKras* on cell autonomous signaling, we isolated and cultured ductal cells from wild type (WT), *cKras*^*Low*^*, cKras*^*Mod*^, and *cKras*^*High*^ mice four days after mice were given tamoxifen (Fig. 5c, d). Cell lysates were prepared after 1 week in culture and subjected to Ras-GTP pull down. Western analysis confirmed a significant increase in Ras-GTP (Ras activity) in *cKra*s^*Mod*^ and *cKras*^*High*^ mice similar to what is observed with whole tissue lysates from these mice pancreata (Fig. 5e, f). We also analyzed total K-Ras levels and observed a significant increase in total K-Ras levels in *cKras*^*High*^ versus *cKras*^*Mod*^ mice. While we had shown increased Ras-GTP as a function of increased percentage of recombined cells, we wanted to determine if this allele also increased the abundance of K-Ras per cell in our model. We probed our cell cultures with CK19 and normalized Ras-GTP and K-Ras to CK19 (Fig. 5g). This normalization indicated a highly significant increase in K-Ras and Ras-GTP in *cKras*^*Mod*^ and *cKras*^*High*^ cultured ducts. To determine if the fold increase in Total K-Ras was due to increased recombination or increased total K-Ras per cell, we calculated the observed fold increase in recombined ducts analyzed in Fig. 1 using IHC (Fig. 5h). Using data from Fig. 1, we calculated an overall average of 3.6-fold increase in recombined ducts in *cKras*^*Mod*^*/cKras*^*Low*^ pancreata. When comparing *cKras*^*High*^*/cKras*^*Mod*^, we observed a 1.43-fold change in overall recombined ducts (Fig. 5h). When comparing *cKras*^*High*^*/cKras*^*Low*^, we observed a 5.7-fold change in overall recombined ducts (Fig. 5h). The fold increases by IHC were used to determine a predicted fold increase by western blot. We then calculated the fold increase observed by western blot using normalized values from total K-Ras/CK19. Using this method, we calculated a 3.9-fold increase in observed fold increase in *cKras*^*Mod*^*/cKras*^*Low*^ ducts. When comparing *cKras*^*High*^*/cKras*^*Mod*^, we calculated a 2.9-fold increase in total K-Ras (Fig. 5h) and *cKras*^*High*^*/cKras*^*Low*^ quantification indicated an 11.6-fold increase in total K-Ras. We compared the observed fold increase by western blot to the predicted fold increase due to recombination analyzed using IHC. When comparing *cKras*^*Mod*^*/cKras*^*Low*^, the observed and predicted fold increase were not significant (3.6 versus 3.9). However, when we compared the predicted and observed fold increase in *cKras*^*High*^*/cKras*^*Mod*^ (2.9 versus 1.43) and *cKras*^*High*^*/cKras*^*Low*^ (11.6 versus 5.7), we observed two-fold increases in observed/predicted fold increase indicating expression of *cKras* increases the abundance of Total K-Ras per cell (Fig. 5h) in *cKras*^*High*^ pancreatic ducts.

### Functional proteomics reveal important pathways expressed in ductal derived PanIN and PDAC

**Fig. 6:**
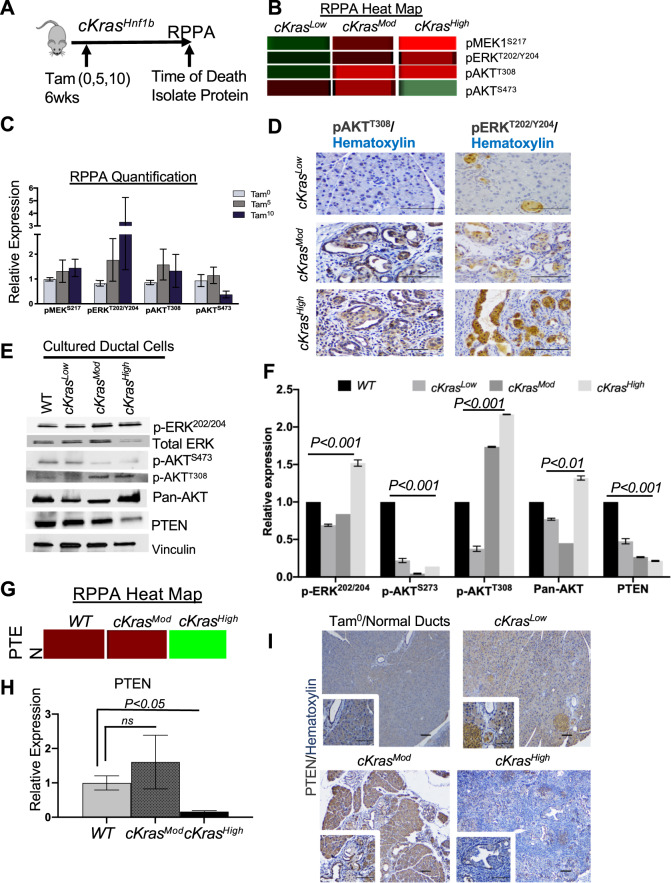
Proteomic analysis of ductal PDAC reveals loss of PTEN. **a** Schematic and (**b**) RPPA heat map data showing increased pMEK^S127^, pERK^T202/Y204^, pAKT^T308^, and decreased expression of pAKT^S273^ in lysates from *WT, cKras*^*Mod*^, and *cKras*^*High*^ pancreata. **c** Quantification of RPPA relative expression data. **d** IHC analysis of pAKT^T308^ and pERK^T202/Y204^ in WT*, cKras*^*Mod*^ and *cKras*^*High*^ pancreata confirms RPPA data that pAKT^T308^ and pERK^T202/Y204^ are significantly increased in *cKras*^*Mod*^ and *cKras*^*High*^ pancreata relative to *cKras*^*Low*^. **e** Western blot analysis of downstream signaling pathways in WT*, cKras*^*Low*^*, cKras*^*Mod*^, and *cKras*^*High*^ cultured pancreatic ducts. **f** Quantification of western blot data normalized to vinculin. **g** Heatmap and (**h**) quantification for PTEN relative expression in WT, *cKras*^*Mod*^, and *cKras*^*High*^ mice. (**i**) IHC staining for PTEN of pancreata from *cKras*^*Low*^*, cKras*^*Mod*^, and *cKras*^*High*^ mice (Zoomed in ×40 magnification images in inset). PTEN expression in normal ducts can be seen in *cKras*^*Low*^*, cKras*^*Mod*^, and *cKras*^*High*^ mice. Scale bars are 50 µM.

We employed a large-scale functional proteomics platform using Reverse Phase Protein Array (RPPA) at MDAnderson Cancer Center. This functional proteomics approach characterizes protein expression and post translational modifications for over 400 proteins. Three samples each of WT, *cKras*^*Mod*^, and *cKras*^*High*^ pancreata were analyzed (Fig. 6). Figure 6a, b and c shows a representative diagram of the experiment (Fig. 6a) and heat map analyses of proteins with high expression (red) versus low expression (green) in analyzed representative samples. Proteomic analysis yielded data regarding elevated MAPK and PI3K/PTEN/AKT pathways in transforming ducts, as pMEK^S127^, pERK^T202/Y204^, and pAKT^T308^ were elevated in *cKras*^*Mod*^ and *cKras*^*High*^ pancreata relative to WT animals (Fig. 6b, c). Using our in vitro model, we analyzed downstream signaling in WT*, cKras*^*Low*^*, cKras*^*Mod*^, and *cKras*^*High*^ cultured ducts. Similar to what we observed in RPPA samples, we observed elevated pERK^202/204^ and elevated pAKT^T308^ but observed a decrease in pAKT^S473^ in *cKras*^*Mod*^ and *cKras*^*High*^ ducts compared to *cKras*^*Low*^ cultured ducts (Fig. 6e, f).

Our RPPA data revealed a highly significant decrease in PTEN expression in *cKras*^*High*^ tissue compared to WT (Fig. 6f, g and h). As PTEN has previously been shown to be highly expressed in pancreatic ducts and centroacinar cells and inhibits IPMN (49, 50), we probed for PTEN expression, which confirmed high levels of PTEN in *cKras*^0^ and *cKras*^*Low*^ ducts; however, we observed a significant reduction in PTEN in *cKras*^*Mod*^ and *cKras*^*High*^ PanIN and PDAC (Fig. 6i). These results were interesting as PTEN has been previously shown to inhibit pAKT^T308^, indicating cell autonomous signaling mechanisms are present in cultured ducts with differences in levels of total K-Ras [49, 50]. To determine if PTEN reduction was an early event in ductal cell transformation, we probed for PTEN in our duct culture system which confirmed a significant reduction in PTEN in *cKras*^*Mod*^ and *cKras*^*High*^ cultured ducts (Fig. 6e–g).

### AKT inhibitor MK-2206 reduces ductal derived PDAC in *cKras*^*High*^ mice

**Fig. 7:**
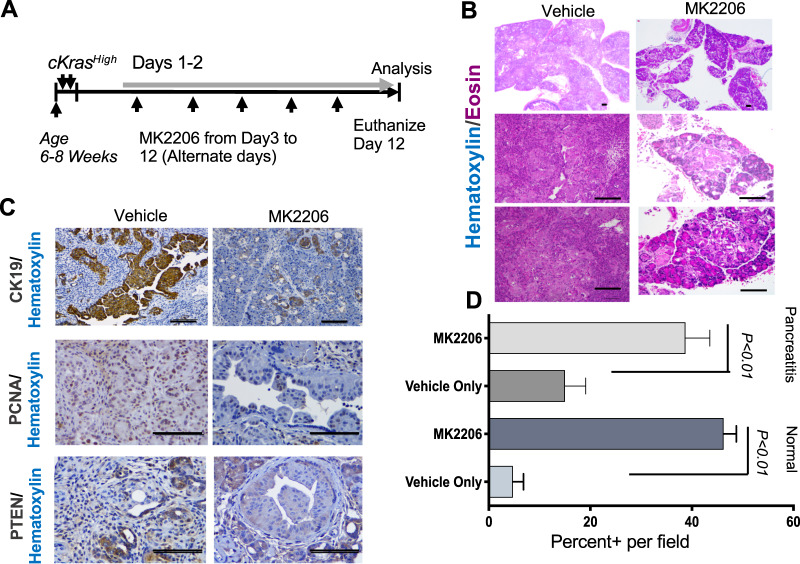
Kras signaling through AKT is important for ductal derived PDAC. **a** Schematic of experimental setup to study the role of AKT in *cKras*^*High*^ mice. **b** Representative H&E sections (×4 and ×20 magnification) show a significant reduction in PDAC in *cKras*^*High*^ pancreata from mice treated with MK2206 relative to vehicle control (*n* = 5 mice analyzed per treatment). **c** CK19, PCNA, and PTEN IHC in vehicle control versus MK2206 treated mice. We observe a reduction in IHC staining for CK19 and PCNA. In resistant advanced PanIN, we observe sustained loss of PTEN. **d** Quantification of normal and pancreatitis percent positive per ×20 field quantified (*n* = 5 per mouse). We observe a significant increase in normal pancreas in MK2206 treated mice. While we do not observe PDAC in MK2206 mice, we do observe significant pancreatitis, indicating sustained inflammation even in the setting of MK2206 treatment. Scale bars are 50 μM. Statistical analysis was performed using a student’s test, Prism GraphPad software.

The role of PI3K/AKT signaling as a potent driver of PDAC has been studied in transgenic mice [50]. As we observed reduced PTEN and elevated pAKT^T308^ in *cKras*^*Mod*^ and *cKras*^*High*^ tissue and primary ductal cultures, we sought to determine if signaling through pAKT^T308^ is required for aggressive ductal derived PDAC. Using MK2206 AKT inhibitor, we treated *cKras*^*High*^ mice every other day and euthanized at day 12 to compare with vehicle treated mice (Fig. 7a). Histologic analysis revealed a significant increase in normal pancreatic area and reduced expression of CK19 in MK2206 treated mice compared to vehicle treated mice (Fig. 7b–d). While we did observe a significant reduction in PDAC (Fig. 7d), we observed sustained pancreatitis in MK2206 treated mice (Fig. 7d). In areas where we observed advanced PanIN, we observed reduced expression of PCNA (Fig. 7c). Notably, resistant neoplastic epithelium had sustained PTEN loss, indicating these lesions were resistant to AKT inhibition and loss of PTEN is important for cuboidal ductal cell to PanIN and PDAC transition. (Fig. 7c).

## Discussion

PDAC remains a highly lethal malignancy. Studying cellular origins of cancer and understanding mechanisms and pathways that drive aggressive tumor formation are important for devising targeted early and late therapeutics for patients. In the context of ductal adenocarcinoma of the pancreas, recent mouse models have shown that pancreatic ductal cells can serve as the cell of origin of PDAC when *Kras* is mutated and, from the onset of oncogenic *Kras*^*mut*^ expression, *Trp53, Fbw7, or Pten* are genetically deleted, or a gain of function mutant of *Trp53* is expressed, using *Hnf1b:CreER*^*T2*^*, Sox9:Cre*^*ER*^*, or Ck19:Cre*^*ER*^ driver lines [4, 17, 22, 41, 42]. As adult pancreatic epithelial cells are refractory to invasive PDAC formation when expressing physiologic levels of *Kras* without loss of tumor suppressor gene and previous manuscripts have shown that *cKras* can produce PDAC from acinar cells [19, 51], we employed the *cKras* allele to study PanIN, IPMN, or and PDAC arising in adult pancreatic ductal cells.

In our genetic system, we have shown we can recombine mutant *cKras* as a function of tamoxifen dosage [40]. Recent sequencing data have shown in human PanIN that when PanIN1, −2, and −3 are sequenced, the percentage of mutant *Kras* positive cells significantly increases when comparing early and advanced PanIN [39]. In this context, we have now characterized a model highly relevant to what is observed in human PanIN, where we increase the percentage of recombined ducts as a consequence of tamoxifen dosage. We have used sophisticated lineage tracing to define the percentage of recombined ductal cells in a dose dependent manner, mimicking what is observed in human PanIN. Notably, in human PanIN, *Trp53* mutations are not detected in PanIN-1 or −2 lesions; only in PanIN-3. In this new model, we observe PDAC arising in the setting of early and advanced PanIN in *cKras*^*Mod*^ mice. *cKras*^*Mod*^ displayed the entire spectrum of PanIN including early PanIN lesions with abundant mucin. Existing ductal cell of origin mouse models have reported PDAC formation primarily through advanced PanIN with limited early PanIN as well as atypical flat lesions (Table 1). In *cKras*^*High*^ mice, PDAC developed mainly through advanced PanIN. Neoplastic ducts also had invasive cells invading into apical duct lumens, which aggravated tumor progression.

**Table 1:** Genetic engineered mouse models of PDAC arising in mature pancreatic duct cells.

Gene promoter	Tumor suppressor loss/mutation	Pancreas histology	Reference
*CK19*^*CreERT*^*; LSL-Kras*^*G12D*^	No	Mostly normal pancreas with scattered PanIN lesions	Kevin C Ray, et al. PLoS One, 2011.
*Hnf1bCre*^*ERT2*^*; Kras*^*G12D*^*, TP53*^*R172H/R172H*^	Yes	Development of invasive PDAC through High grade PanIN lesions	Bailey JM, et al., Oncogene, 2016. **35**(32): p. 4282–8
*Sox9*^*CreER*^;* Kras*^*LSL-12D*^; *Trp53*^*flox/flox*^	Yes	High Grade PanIN lesions and PDAC	Lee AYL, et al., Gut. 2018
*Ck19-CreER; Kras* ^*LSL-G12D/wt*^*; Fbw7*^*f/f*^	Yes	Development of invasive PDAC through non mucinous tubular lesions.	Ferreira RMM, et al., Cell Reports. 2017
*Sox9CreER*^*T2*^; *Kras*^*G12D*^*; Pten*^*flox/flox*^; *R26R*^*YFP*^	Yes	Pancreatobilliary type IPMN and invasive PDAC	Kopp JL, et al., Gastroenterology. 2018
*CK19*^*CreERT2*^*; LSL-KrasG12D; R26R*^*EYFP*^			
*Sox9*^*CreERT2*^*; LSL-Kras*^*G12D*^*; R26R*^*EYFP*^			
*with pancreatic duct ligation (chronic pancreatitis)*	No	PDAC development through flat epithelial precursors. Few PanIN lesions observed.	Shi C, et al. CMGH, 2019.

We also found that as a higher number of cells expressed mutant *cKras* there was an overall increase PanIN, PDAC, fibrostroma, and inflammation. In the context of ductal cells as origin of PDAC, a recent study presented PDAC arising in the setting of duct obstruction after pancreatic duct ligation which led to neoplasia and PDAC from duct cells expressing *Kras*^*mut*^ from endogenous locus [41]. In our model, we show development of pancreatitis which increases as we enhance the abundance of intrapancreatic ductal neoplasia. It can be noted that, especially in our *cKras*^*High*^ model, significant duct obstruction may be present from advanced PanIN, which promotes widespread pancreatitis.

We report proteomics analysis performed on pancreatic tissue obtained from WT*, cKras*^*Mod*^, and *cKras*^*High*^ mice. RPPA analysis revealed a spectrum of differentially activated MAPK and PI3K. Similar to previous reports, we observed ductal epithelial cells lacked expression of p21 and were highly proliferative (Supplementary Fig. 4). In this model we see loss of tumor suppressor PTEN, a well-established tumor suppressor in human cancers. The role of PTEN in pancreatic ductal malignancy has been studied in recent years. Notably, loss of *Pten* and expression of *Kras* promotes an IPMN-PDAC phenotype in *Sox9* expressing ductal epithelium [52]. In our model, we observed PanIN-PDAC and did not observe IPMN, indicating potential divergent mechanisms of ductal cell transformation in the context of *PTEN* gene expression loss as an initiating event or occurring spontaneously during tumorigenesis. Future studies using this model will aid in understanding how elevated levels of K-Ras in murine PDAC decreases PTEN levels [49, 52–54]. Using tissue lysates and our in vitro cell cultre platform, we analysed AKT phosphorylation. We observed increased phosphorylation of AKT at Threonine site at amino acid 308 (pAKT^T308^) whereas pAKT^S473^ was decreased. AKT is known to be fully active when phosphorylated at both these sites. However, recent emerging data has shown pAKT^T308^ is a more potent driver and marker of aggressive disease in non –small cell lung cancer and pAKT^T308^, rather than pAKT^S473^, correlates with AKT downstrem effectors [55]. In our model, while we have not completely interrogated downstrem pathways of AKT, we did employ a pan AKT inhibior (MK2206), which significanlty reduced aggressive PDAC in *cKras*^*High*^ mice. Existing neoplastic ducts were seen to be less proliferative, but remained associated with pancreatitis. Our results and previous work [56] show the importance of studying both AKT phosphoryaltion sites and its effectors in patient tumor samples for better prognosis and to develop targetted therapies. In summary, our mouse model provides an important new system to study how levels of total K-Ras and Ras activity drive intraductal lesions including PanIN and PDAC. This system will be very relevant for studying novel therapeutic strategies in the context of Ras signaling and this model provides an important Ras rheostat tool to study not only underlying mechanisms of PDAC arising in pancreatic ductal epithelium, but also better understanding how to calibrate therapeutics.

## Methods

### Transgenic mouse models

*Hnf1bCreER*^*T2*^ mice were purchased form Jackson Laboratories. Transgenic mice with CAG-*lox-GFP-stop-lox-Kras*^*G12V*^ were received from Craig Logsdon, MD Anderson Cancer Center, Houston, TX [19]. Strains of *Hnf1bCreER*^*T2*^ mice were crossed with *cLGL-KRAS*^*G12V*^ to generate *cKras*^*Hnf1b/+*^ mice and obtain mutant c*Kras* expression in adult pancreatic ductal cells. These mice express GFP in whole body and lose GFP after Cre mediated recombination. Mice were genotyped by PCR or Transnetyx. Mice were injected with tamoxifen at an age of 6–8 weeks and an *n* = 10–12 mice was evaluated for each tamoxifen dose. To achieve different levels of mutant c*Kras* in ductal cells, mice were injected with 1 mg tamoxifen (Sigma, T5648) subcutaneously one day (1 mg dose), 5 mg tamoxifen for 1 day (5 mg dose), and 5 mg tamoxifen for 2 consecutive days (10 mg dose). All pancreatic pathologies in genetically engineered mice and humans were classified by a pathologist as has been classified and described previously [57, 58]. PanIN were classified on a two-tier grade based on cognate human classification system [59]. All animal studies were approved by the Animal Care and Use Committees at University of Texas Health Science Center at Houston.

### Ras activity pull down assay

Ras activity in the *cKras*^*Hnf1b/+*^ mice pancreas samples was performed using the Active Ras pull down and detection kit (Thermofisher Scientific). Up to 30 mg of tissue (fresh or frozen at −80 °C) was washed with 1X cold PBS, cut into smaller pieces and homogenized in 1 ml lysis buffer (Cell Signaling Technology, 9803 S) containing protease inhibitor cocktail (Roche, 4693159001). Homogenized tissue was placed on ice for 30 min followed by sonication for 2 min with 10 s on/off cycle. The lysate was centrifuged for 20 min at 10,000 × *g* at 4 °C in a micro centrifuge. The pellet was discarded, and the lysate was used for protein estimation by BCA method. Aliquots of lysates were saved for further quantification of total Ras or protein loading controls by immunoblotting and a 500 μg equivalent of lysate protein were incubated for 45 min at 4 °C, with beads coated with Raf1-RBD provided with the kit. Beads were then washed 3 times with ice-cold lysis buffer, and bound protein was eluted for 15 min with Laemmli sample buffer that had been preheated to 95 °C and analyzed by immunoblotting for Ras following Western blot analysis.

### Western blot analysis

Tissue extracts were prepared using cell lysis buffer (Cell signaling #9803) with protease inhibitor cocktail tablets (Cell signaling #5871). Tissue extracts were prepared using cell lysis buffer (Cell signaling #9803) with protease inhibitor cocktail tablets (Cell signaling #5871). Protein concentrations were determined by BCA method. A 20 µg of protein was analyzed using SDS page. The proteins were transferred by semi dry method on to the nitrocellulose membrane using Trans blot Turbo Transfer (Bio-Rad). Membranes were blocked using 5% skimmed milk and then incubated with primary antibodies overnight at 4 °C. Next day the membrane was washed 4 times with TBST buffer and incubation with the respective HRP-conjugated secondary antibody used at 1:5000 for 1 h. Membranes were washed and developed using Clarity^TM^ Western ECL Substrate (Bio-Rad **#**1705061). Primary antibodies used in this study are described in Supplementary Table 1.

### Primary pancreatic duct culture

Pancreatic ducts were cultured as defined previously [60, 61] from *cKras*^*0*^ *(WT), cKras*^*Low*^*, cKras*^*Mod*^, and *cKras*^*High*^ mice four days after tamoxifen was administered. Briefly, Pancreas was collected, minced to 1 mm pieces and digested for 30 min at 37 °C in digestive solution (0.1% soybean trypsin inhibitor and 0.1% Collagenase). Cells were filtered through 40 µm filter, washed additional two times with culture medium and plated on collagen coated plates in complete medium DMEM/F12 (Life Technologies 11330-032) 500 mL, Penicillin‐streptomycin (100×; Life Technologies 15140-122) 5 mL, 1×Nu-serum IV (BD Biosciences 355104) 25 mL, 5% Bovine pituitary extract (3 mg/mL; BD Biosciences 354123) 4.2 mL, 25 μg/mL ITS + Premix (BD Biosciences 354352) 2.5 mL, Epidermal growth factor (100 μg/mL; BD Biosciences 354001) 100 μL, 20 ng/mL Cholera toxin (1 mg/mL; Sigma-Aldrich C8052) 50 μL, 100 ng/mL3,3′,5-Triiodo-L-thyronine (50 μM; Sigma-Aldrich T2877)50 μL, 5 nM Dexamethasone (100 mM; Sigma-AldrichD1756) 5 μL, 1 μM D-Glucose (Sigma-Aldrich G5400) 2.5 g 4.7 mg/mL, Nicotinamide (Sigma-Aldrich N3376) 0.66 g 1.22 mg/mL and Soybean trypsin inhibitor (type I; Sigma-Aldrich T6522) 50 mg 0.1 mg/mL. The cultures grew to confluency in one week and fibroblast contamination was reduced using differential trypsinization method. Cell lysate were collected and equal amounts of protein were subjected to Western analysis.

### Immunofluorescence and immunohistochemistry

Tissues were fixed in 4% paraformaldehyde, processed according to standard protocols and embedded in paraffin. The unstained sections were baked at 60 °C for 30 min. The sections were deparaffinized with Histoclear and rehydrated. Antigen retrieval was performed using heat-mediated microwave methods and an antigen unmasking solution (Vector Laboratories, H-3300) was used for all antibodies. All sections were blocked in 10% FBS in PBST and primary antibodies were incubated overnight at 4 °C. Secondary antibodies were used at 1:500 and incubated at room Temperature for 2 h for IF and 30 min for IHC. For IF, slides were stained with IHC-Tek Dapi counterstain solution (IHC World, IW-1404) and mounted in fluorescence mounting medium (Dako, S3023). For IHC, Vectastain Elite ABC kit (Vector Laboratories, PK-6100) and DAB Peroxidase (HRP) Substrate kit (Vector Laboratories, SK-4100) were used. Primary antibodies used in this study are described in Supplementary Table 1.

### Functional proteomics/RPPA analysis

Differentially tamoxifen dosed *cKras*^*Hnf1b/+*^ mice were monitored after tamoxfien injection and were euthanized at their humane timepoint. Pancreas tissue was collected an lysed using lysis buffer 1% Triton X-100, 50 mM HEPES, pH 7.4, 150 mM NaCl, 1.5 mM MgCl2, 1mMEGTA, 100 mM NaF, 10 mM Na pyrophosphate, 1 mM Na3VO4, 10% glycerol, containing freshly added protease and phosphatase inhibitors (Sigma Aldrich,St. Louis, MO) and protein concentration was measured by bicinchoninic acid (BCA) method. Protein concentrations of samples were adjusted to 1 mg/ml with lysis buffer. Cell lystes were serially diluted twofold for 5 dilutions (from undiluted to 1:16 dilution) and arrayed on nitrocellulose-coated slides in an 11 × 11 format. Samples were probed with antibodies by tyramide-based signal amplification approach and visualized by DAB colorimetric reaction. The slides were analyzed and protein expression quantitated with the use of Array-Pro Analyzer. All the data points were normalized for protein loading and transformed to linear value, designated as “Normalized Linear”.

### MK-2206 treatment

Six to eight-week-old *cKras*^*Hn1b/+*^ mice were administered 5 mg of tamoxifen on days 1 and 2 (total 10 mg dose). MK-2206 (Selleckchem) was given on alternate days starting on Day 3 through Day 12. MK-2206 was administered using intraperitoneal injection at a dosage of 120 mg/kg.

### ImageJ analysis

Area of pancreatitis was measured using imageJ (http://imagej.nih.gov/ij/) software. Five randomly chosen fields of H&Es of each pancreatic section were selected for quantification. Pancreatitis was calculated as percentage of total pancreatic area. Color deconvolution plugin was used to isolate pancreatitis versus normal pancreatic acinar cell areas. 5 fields per slide were selected for quantification and 5 mice were analyzed per tamoxifen dose. For Collagen fiber quantification, Masson’s trichrome stained pancreatic sections were used. The color deconvolution plugin of ImageJ software was used to quantify the collagen area in five randomly chosen fields of each pancreatic section as has been previously described [62]. Fibrosis was calculated as percentage of total pancreatic area. In total, 5 fields per slide were selected for quantification and 5 mice were analyzed per tamoxifen dose.

## Supplementary information

Supplemental Figures and Table
