# Correction to: Kras mutation rate precisely orchestrates ductal derived pancreatic intraepithelial neoplasia and pancreatic cancer

**DOI:** 10.1038/s41374-021-00615-4

**Published:** 2021-06-02

**Authors:** Kanchan Singh, Melissa Pruski, Rachael Bland, Mamoun Younes, Sushovan Guha, Nirav Thosani, Anirban Maitra, Brooks D. Cash, Florencia McAllister, Craig D. Logsdon, Jeffrey T. Chang, Jennifer M. Bailey-Lundberg

**Affiliations:** 1 Division of Gastroenterology, Hepatology and Nutrition, Department of Internal Medicine, McGovern Medical School, The University of Texas Health Science Center, Houston, TX USA; 2 Kings College London, Department of Pharmacology, London, UK; 3 Department of Pathology and Laboratory Medicine, McGovern Medical School, The University of Texas Health Science Center, Houston, TX USA; 4 Department of Translational Molecular Pathology, Division of Pathology and Laboratory Medicine, The University of Texas MD Anderson Cancer Center, Houston, TX USA; 5 Department of Gastrointestinal Medical Oncology, The University of Texas MD Anderson Cancer Center, Houston, TX USA; 6 Department of Cancer Biology, The University of Texas MD Anderson Cancer Center, Houston, TX USA; 7 Department of Integrative Biology and Pharmacology, McGovern Medical School, The University of Texas Health Science Center, Houston, TX USA; 8 Department of Anesthesiology, McGovern Medical School, The University of Texas Health Science Center, Houston, TX USA

**Keywords:** Cancer models, Mechanisms of disease

Correction to: *Laboratory Investigation*

10.1038/s41374-020-00490-5

The article Kras mutation rate precisely orchestrates ductal derived pancreatic intraepithelial neoplasia and pancreatic cancer, written by Kanchan Singh, Melissa Pruski, Rachael Bland, Mamoun Younes, Sushovan Guha, Nirav Thosani, Anirban Maitra, Brooks D. Cash, Florencia McAllister, Craig D. Logsdon, Jeffrey T. Chang and Jennifer M. Bailey-Lundberg, was originally published electronically on the publisher’s internet portal on the 2^nd^ October 2020 without open access. With the author(s)’ decision to opt for Open Choice the copyright of the article changed on the 6^th^ May 2021 to © The Author(s) 2021 and the article is forthwith distributed under a Creative Commons Attribution 4.0 International License, which permits use, sharing, adaptation, distribution and reproduction in any medium or format, as long as you give appropriate credit to the original author(s) and the source, provide a link to the Creative Commons licence, and indicate if changes were made. The images or other third party material in this article are included in the article’s Creative Commons licence, unless indicated otherwise in a credit line to the material. If material is not included in the article’s Creative Commons licence and your intended use is not permitted by statutory regulation or exceeds the permitted use, you will need to obtain permission directly from the copyright holder. To view a copy of this licence, visit http://creativecommons.org/licenses/by/4.0/.

