# Supplementary material for: Kras mutation rate precisely orchestrates ductal derived pancreatic intraepithelial neoplasia and pancreatic cancer
Source: Lab Invest. 2020 Oct 2;101(2):177–92. doi: 10.1038/s41374-020-00490-5 (PMC8172380; doi:10.1038/s41374-020-00490-5)
Supplement: Supplementary file 1 — Supplemental Figures and Table [file 41374_2020_490_MOESM1_ESM.pdf]

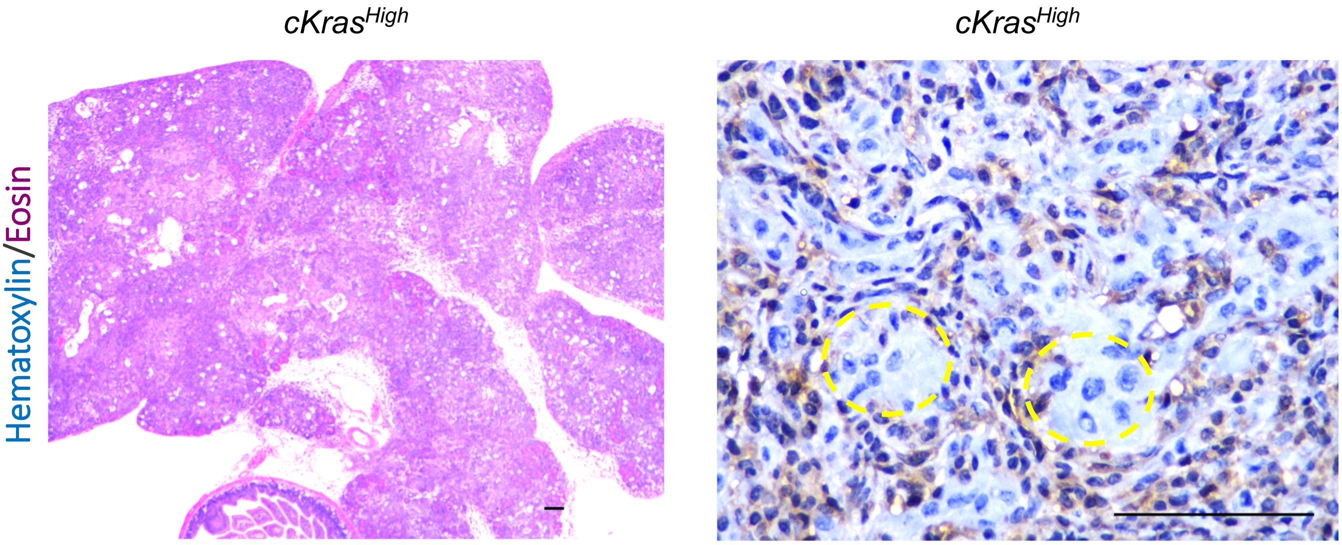

Supplemental Figure 1. Expression of *cKras* in greater than 90% of pancreatic ducts generates poorly differentiated PDAC. H&E analysis confirms abundant PDAC and IHC staining for GFP reveals loss of GFP/recombination of $cKras^{High}$ in PDAC tumors. Scale bars are 50uM.

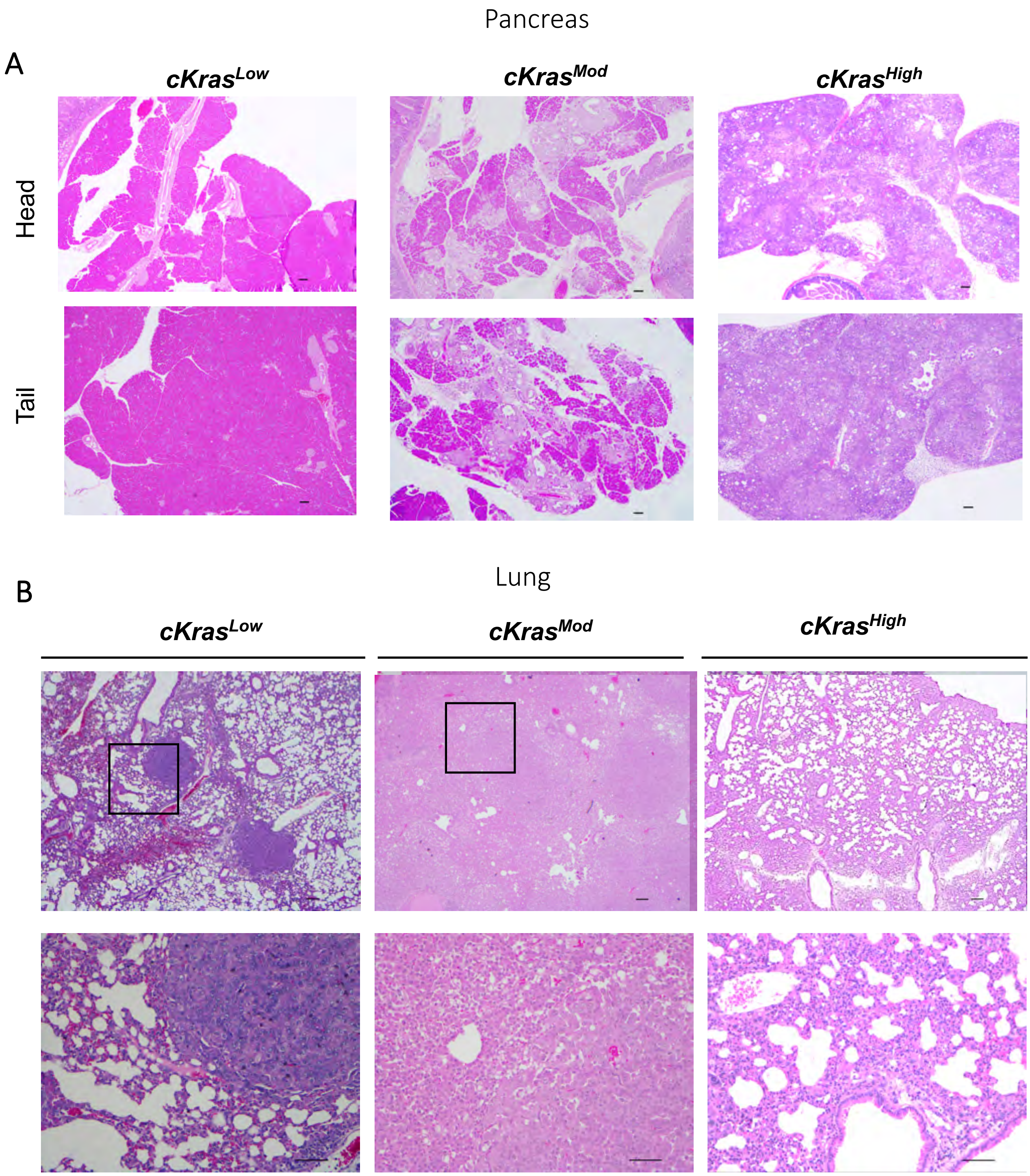

Supplemental Figure 2. Expression of *cKras* in pancreatic ducts promotes PanIN and PDAC in head and tail. A. H&E analysis confirms PanIN and PDAC in head and tail regions of the pancreas in $cKras^{Mod}$ and $cKras^{High}$ mice. B. Lung pathology of *$cKras^{Hnf1b/+}$ mice.* *$cKras^{Low}$* (adenoma), *$cKras^{Mod}$* (adenocarcinoma) and *$cKras^{High}$* (inflammation) mice. Scale bars are 50uM.

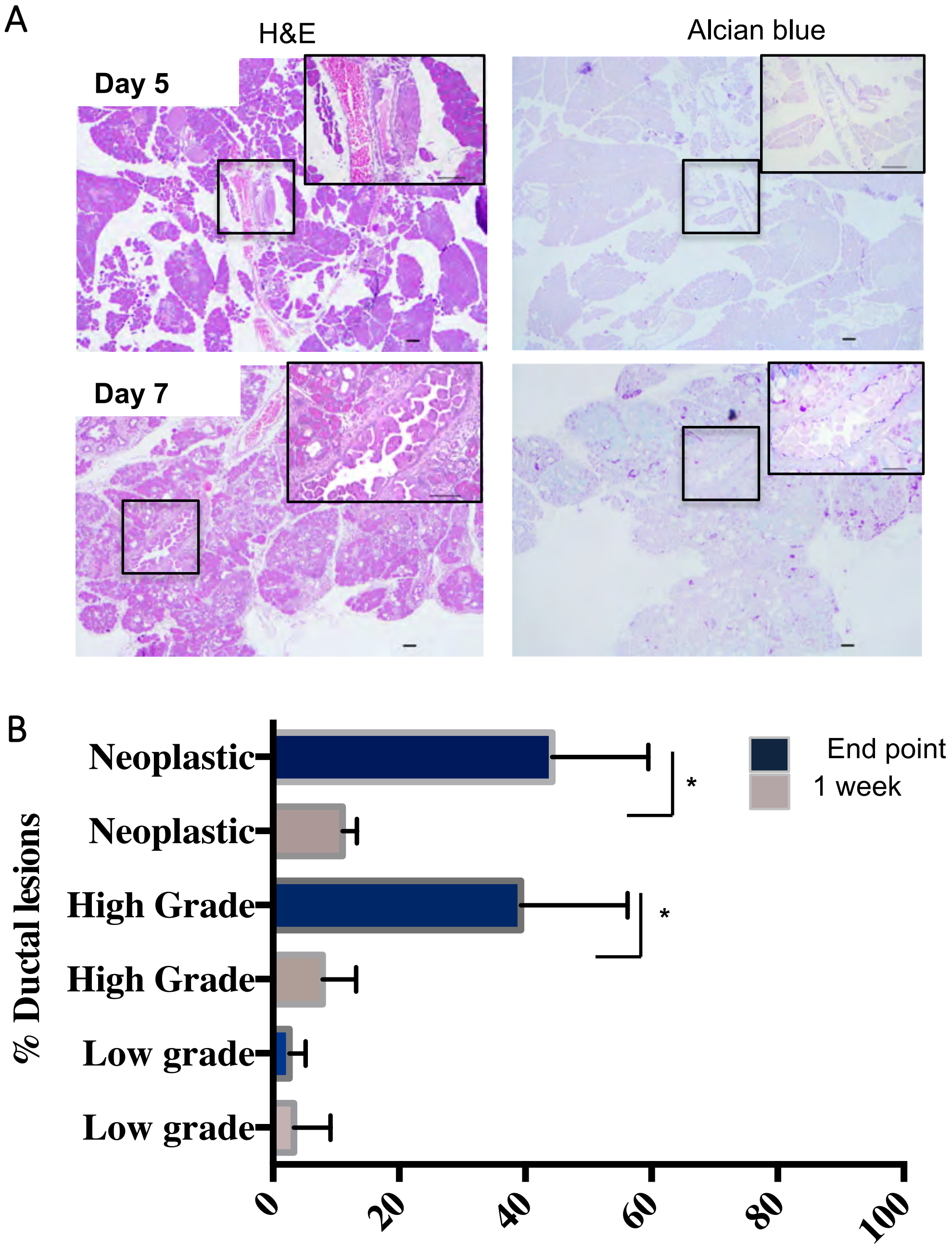

Supplemental Figure 3, Related to Figure 3. *cKras*^{High} mice generate PDAC in the absence of early PanIN. (A) *cKras*^{High} mice progress to PDAC through advanced PanIN lesions (inset higher magnification). Early time points day 5 and day 7. H&E showing advanced PanIN and adjacent panels are Alcian Blue staining showing lack of mucin in these lesions. Scale bar is 50um.

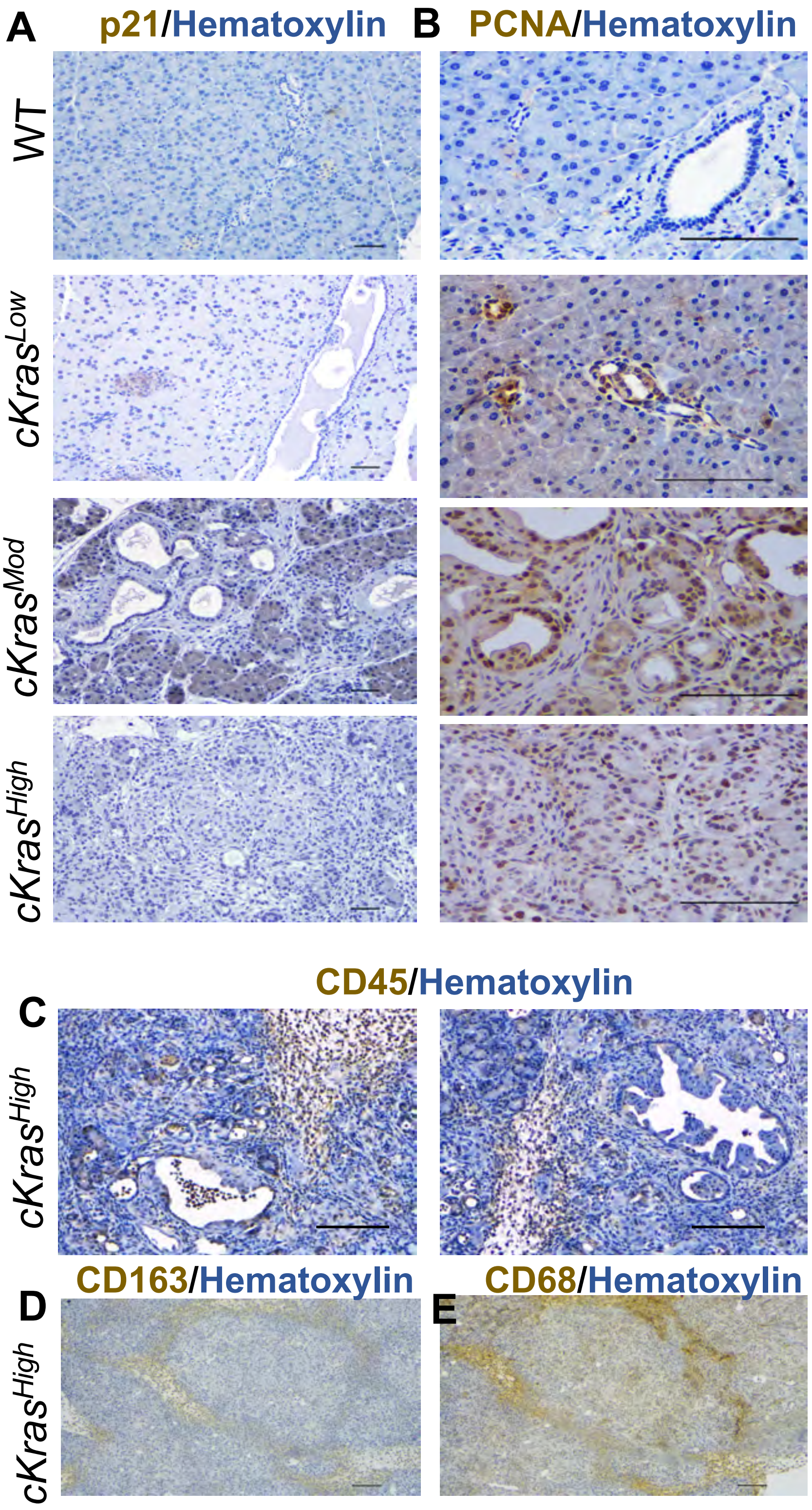

Supplemental Figure 4: Ductal derived PanIN and PDAC do not express the senescent marker p21. (A) IHC data showing lack of p21 labeling in ductal regions of *cKras*^{Low}, *cKras*^{Mod} and *cKras*^{High} mice. (B) PCNA labeling confirms proliferative ductal cells in *cKras*^{Low}, *cKras*^{Mod} and *cKras*^{High} mice. (C) CD45 immunolabeling in *cKras*^{High} reveals abundant lymphocyte or immune infiltration in ductal derived PDAC. (D, E) CD163 and CD68 immunolabeling further subtype immune infiltration to show M2 macrophage are present in *cKras*^{High} ductal derived PDAC. Scale bars are 50uM. Three slides per mouse were stained with the described antibodies. Representative images are shown. Scale bars are 50um.

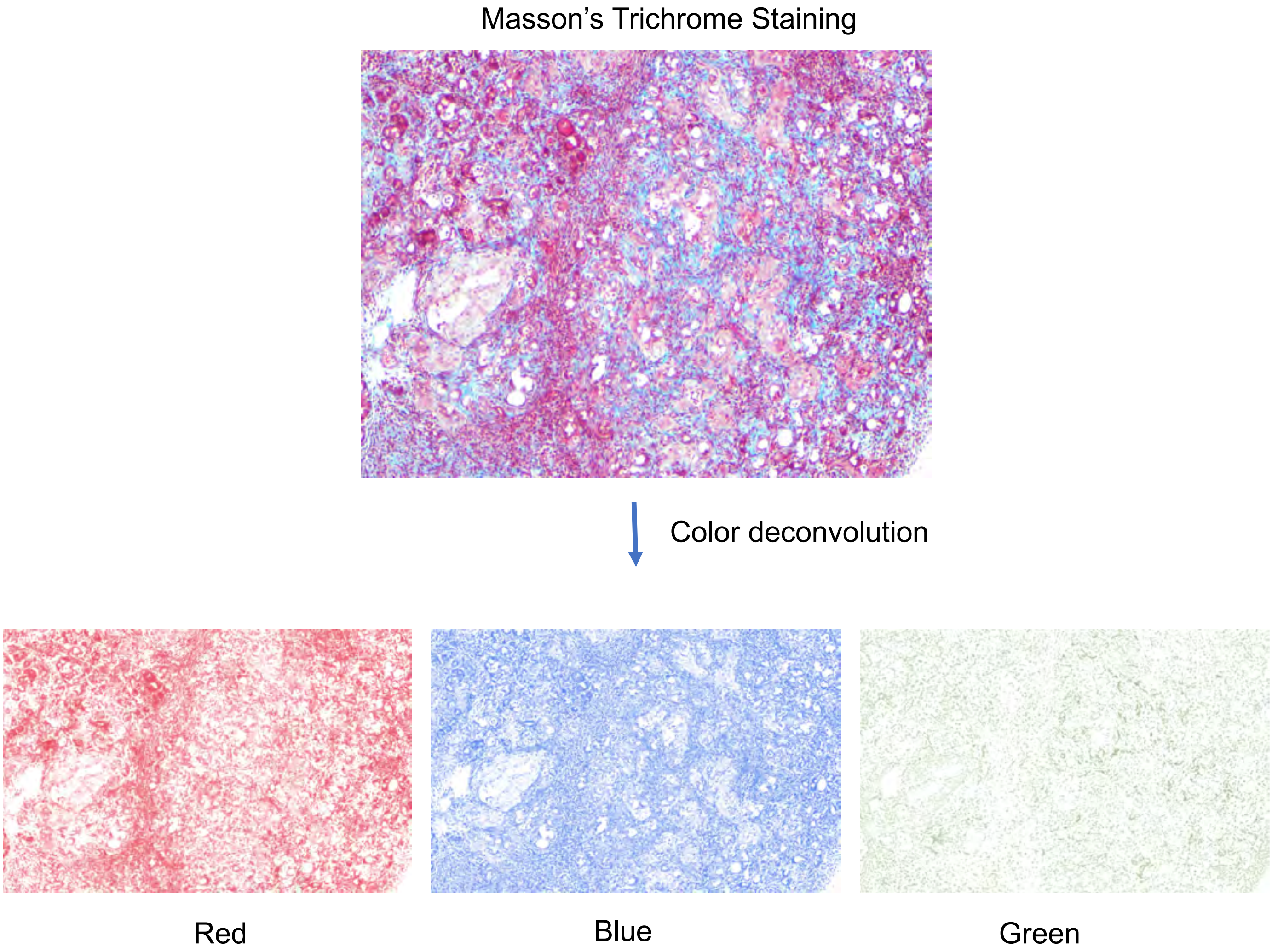

Supplemental Figure 5: Representantive images of deconvolution using imageJ software to quantify collagen composition in trichrome stained images. For Collagen fiber quantification, Masson's trichrome stained pancreatic sections were used. The color deconvolution plugin of ImageJ software was used to quantify the collagen area in five randomly chosen fields of each pancreatic section. Fibrosis was calculated as percentage of total pancreatic area. Three mice per group were quantified.

Supplementary Table 1. List of Antibodies used in the study

Antibodies	Dilution IHC	Dilution Western blotting	Species	Catalogue no	Source
Ras	X	1:200	Mouse	16117	Thermofisher Scientific
KRT19	1:100	1:1000	Rat	TROMA III	DSHB
Phospho-p44/42 MAPK (Erk1/2) (Thr202/Tyr204)	1:100	1:1000	Rabbit	4370	Cell Signaling Technology
p44/42 MAPK (Erk1/2)	1:100	1:1000	Rabbit	4695	Cell Signaling Technology
Phospho-Akt (Thr308)	1:100	1:1000	Rabbit	38449	Cell Signaling Technology
Phospho-Akt (S473)	1:100	1:1000	Rabbit	4060	Cell Signaling Technology
Akt (pan)	1:100	1:1000	Rabbit	4685	Cell Signaling Technology
Phospho-EGF Receptor (Tyr1068)	1:100	1:1000	Rabbit	3777	Cell Signaling Technology
PTEN	1:100	1:1000	Rabbit	9188	Cell Signaling Technology
GFP	1:200	1:1000	Mouse	Sc-9966	Santa Cruz
PCNA	1:2000	X	Mouse	Pc-10 Ab-29	Abcam
P53	1:100	X	Mouse	Sc-126	Santa Cruz

DSHB- Developmental Studies Hybridoma Bank
